# Supplementary material for: Thermal-bias PCR: generation of amplicon libraries without degenerate primer interference
Source: PeerJ. 2025 Oct 24;13:e20241. doi: 10.7717/peerj.20241 (PMC12558157; doi:10.7717/peerj.20241)
Supplement: Supplemental Information 1 — The PCR model was used to generate reaction profiles using different input values. (A) The PCR model: the amplicon yield in a given thermal cycle is a function of the prior cycle’s amplicon abundance (prev), the maximum theoretical yield of the completed reaction (max), and the inhibitory activity of accumulated products (KD). After a reaction’s max and KD values are obtained by fitting the model to qPCR data, the abundance of the initial template can be calculated (seed). (B) Modeled reaction profiles using different seed values of 0.1, 0.001, or 0.00001 and with invariant max = 1E4 and KD = 1E3. The different seed values shift the positions of the reaction profiles, but the profile shapes remain the same. (C) Using invariant seed, max, and KD values, the maximum possible per-cycle efficiency was set to 100% (2-fold amplification), 95% (1.9-fold amplification), or 90% (1.8-fold amplification). (D) Reactions in which the value of max was reduced in 2-fold increments. (E) Reactions in which the value of KD was reduced in 2-fold increments. (F) Different ratios of max/KD change the reaction profiles and can produce the same yield at a given cycle. Shown are three examples that produce the same yield at the 30th cycle (marked in red): max = 1E4, KD = 1E3 (max/KD = 10); max = 1.83E4, KD = 5E2 (max/KD = 36.6); and max = 7.8E4, KD = 2.5E2 (max/KD = 312). [file peerj-13-20241-s001.pdf]

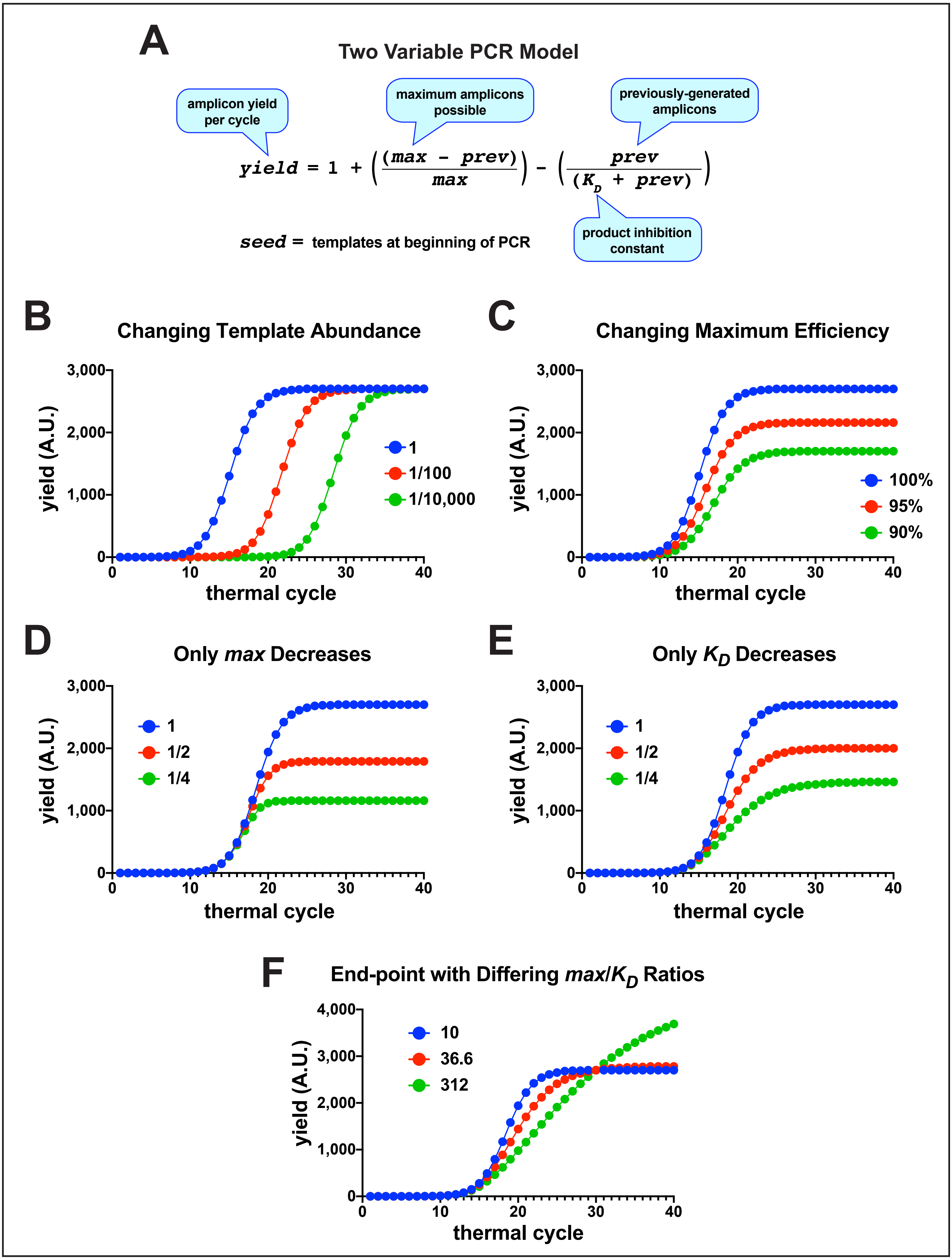
A
Two Variable PCR Model
amplicon yield per cycle
maximum amplicons possible
previously-generated amplicons
yield = 1 + ((max - prev) / max) - (prev / (K_D + prev))
product inhibition constant
seed = templates at beginning of PCR
B
Changing Template Abundance
yield (A.U.)
thermal cycle
1
1/100
1/10,000
C
Changing Maximum Efficiency
100%
95%
90%
D
Only max Decreases
1
1/2
1/4
E
Only K_D Decreases
1
1/2
1/4
F
End-point with Differing max/K_D Ratios
10
36.6
312

S1 Figure. Modeling the influence of *max* and K_D on PCR reaction quality.
The PCR model was used to generate reaction profiles using different input values. **A)** The PCR model: the amplicon yield in a given thermal cycle is a function of the prior cycle's amplicon abundance (*prev*), the maximum theoretical yield of the completed reaction (*max*), and the inhibitory activity of accumulated products (K_D). After a reaction's *max* and K_D values are obtained by fitting the model to qPCR data, the abundance of the initial template can be calculated (*seed*). **B)** Modeled reaction profiles using different *seed* values of 0.1, 0.001, or 0.00001 and with invariant *max* = 1E4 and K_D = 1E3. The different *seed* values shift the positions of the reaction profiles, but the profile shapes remain the same. **C)** Using invariant *seed*, *max*, and K_D values, the maximum possible per-cycle efficiency was set to 100% (2-fold amplification), 95% (1.9-fold amplification), or 90% (1.8-fold amplification). **D)** Reactions in which the value of *max* was reduced in 2-fold increments. **E)** Reactions in which the value of K_D was reduced in 2-fold increments. **F)** Different ratios of *max*/K_D change the reaction profiles and can produce the same *yield* at a given cycle. Shown are three examples that produce the same *yield* at the 30th cycle (marked in red): *max* = 1E4, K_D = 1E3 (*max*/K_D = 10); *max* = 1.83E4, K_D = 5E2 (*max*/K_D = 36.6); and *max* = 7.8E4, K_D = 2.5E2 (*max*/K_D = 312).

Additional model evaluations: PCR amplification profiles reveal reaction quality
Experimental qPCR data of efficient reactions can exhibit *max*/K_D ratios on the order of ~10, so modelling was initiated using a PCR model (S1A) using a fixed 10:1 ratio and the *seed* values were altered to represent differing initial template abundances (S1B). Consistent with our prior characterization of this PCR model [22], altering the *seed* value did not change the shapes of the reaction profiles, but the relative spacing of the data reflected the changes in template abundance. Shifts in reaction curve spacing are the basis of relative template quantification using either this global fitting approach or ΔCq methods.

qPCR protocols may implement additional calibration measurements to determine the per-cycle efficiency by comparing the Cq values of known template dilutions [19,21]. To model the impact of reduced reaction efficiencies on *max* and K_D, forced reductions to per-cycle reaction efficiencies were implemented by changing the '1' in the PCR model to lower values such that the maximum per-cycle yield ranged from 100% (2-fold amplification during the first cycle) to 90% (1.8-fold amplification) (S1C). Expectedly, the modeled data indicated that a reduction in reaction efficiency not only lowered the plateau heights (overall yield) and decreased the maximum slopes, but also caused substantial shifts in the profile positions to later cycles. For example, although the same *seed* values were used for each modeled reaction, a reduction in maximal efficiency to 90% shifted the reaction curve by ~2 cycles (corresponding to a ~4-fold reduction in the apparent template concentration). These data were subsequently fit using the PCR model with the maximum efficiency restored to '1' to evaluate the impact on the *seed*, *max*, and K_D values for each curve. Consistent with ΔCq, a reduction of maximal efficiency to 90% caused the resulting *seed* value for that reaction to lower by ~4-fold (26.7%). Importantly, this distortion was accompanied by a reduction in both the *max* and K_D by ~19% and ~55%, respectively, which caused the *max*/K_D ratio to increase by ~80%. This trend in increasing *max*/K_D ratios continued until the per-cycle

efficiency was reduced to 65%, at which point the algorithm failed to fit. Therefore, even though the *max* and K_D values obtained from a given reaction's fit are influenced by several experimental variables (such as fluorophore response, per-well measurement variations, and measurement technique), the *max*/K_D ratio is an unitless metric of reaction quality that can be used to distinguish changes in reaction performance from *bona fide* changes in template abundance.

To clarify the influence of the *max* and K_D values on reaction profiles, each was varied independently. Reductions to the *max* term in the model lowered the plateau values, yet had a negligible effect on the on the emergence of the data above baseline (no Cq variance) (S1D); whereas reductions to K_D delayed baseline emergence, lowered amplification slopes, and lowered plateau heights (S1E Figure). Because the *max* term represents available resources and the K_D term represents inhibition, this modeling suggests that reaction 'poisoning' is primarily responsible for non-ideal reaction profiles, not reagent limitation.

Endpoint PCR amplicon abundance is frequently used to evaluate PCR efficiencies under different conditions. The molecular mechanisms that stall reactions have not been fully resolved, but it has been proposed that amplicon reannealing eventually outcompetes primer annealing [20]. It is commonly asserted that PCRs become exhausted (plateau) because of reagent depletion; however, the remaining concentrations of primers and dNTPs in exhausted reactions substantially exceed the concentration of generated amplicons [31], and the residual primer concentrations are well within a range that would otherwise support robust amplification (for example, reactions having 500 nM of each primer generate ~100 nM of amplicons). The modeling data presented above suggest that the trajectories of amplicon accumulation toward endpoint values may be more informative of reaction quality than overall yield. To evaluate this idea, reactions were modeled with different *max*/K_D ratios such that the same amplicon abundance was present after 30 cycles, a common end-point protocol (S1F). It can be seen that the amplicon abundance at a given cycle preceding the plateau can be highly variable and dependent on reaction quality. Thus, end-point analyses can be misleading indicators of reaction efficiency. Taken together, this modelling activity revealed that elevated *max*/K_D ratios are telling of underlying problems and that this ratio can be used to comparatively evaluate reaction quality.
