# Supplementary material for: Thermal-bias PCR: generation of amplicon libraries without degenerate primer interference
Source: PeerJ. 2025 Oct 24;13:e20241. doi: 10.7717/peerj.20241 (PMC12558157; doi:10.7717/peerj.20241)
Supplement: Supplemental Information 3 — The V3-V4 region was amplified from E. coli genomic DNA using reaction conditions recommended by the enzyme manufactures. Top panel, SsoFast EvaGreen (Bio-Rad); middle panel, iTaq SYBR Green (Bio-Rad); bottom panel, Q5 (NEB) supplemented with EvaGreen (Jena Bioscience). [file peerj-13-20241-s003.pdf]

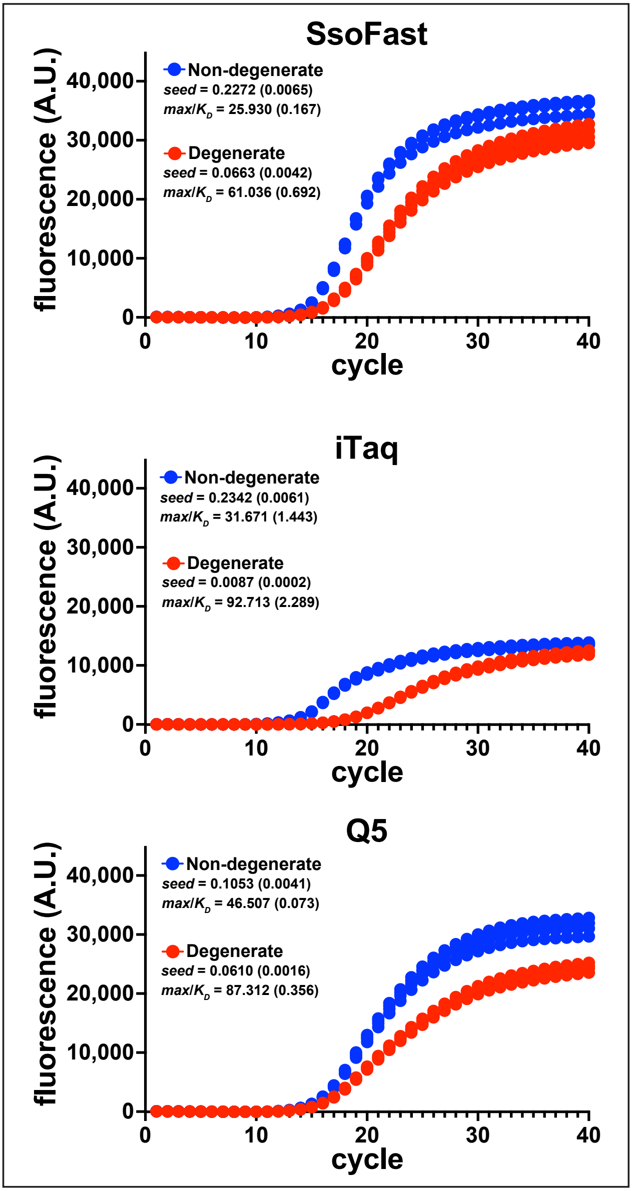

S2 Figure. Different polymerases using degenerate or non-degenerate primers. The V3-V4 region was amplified from *E. coli* genomic DNA using reaction conditions recommended by the enzyme manufactures. Top panel, SsoFast EvaGreen (Bio-Rad); middle panel, iTaq SYBR Green (Bio-Rad); bottom panel, Q5 (NEB) supplemented with EvaGreen (Jena Bioscience).
