# Supplementary material for: Thermal-bias PCR: generation of amplicon libraries without degenerate primer interference
Source: PeerJ. 2025 Oct 24;13:e20241. doi: 10.7717/peerj.20241 (PMC12558157; doi:10.7717/peerj.20241)
Supplement: Supplemental Information 4 — Shown are the nucleotide sequences of the top strands of the ’match’ and ’mismatch’ reporters. Each has a unique restriction site inserted. The mismatch version has four nucleotide changes (red) in the primer-binding regions that are in positions that should be compensated by the degenerate primers. [file peerj-13-20241-s004.pdf]

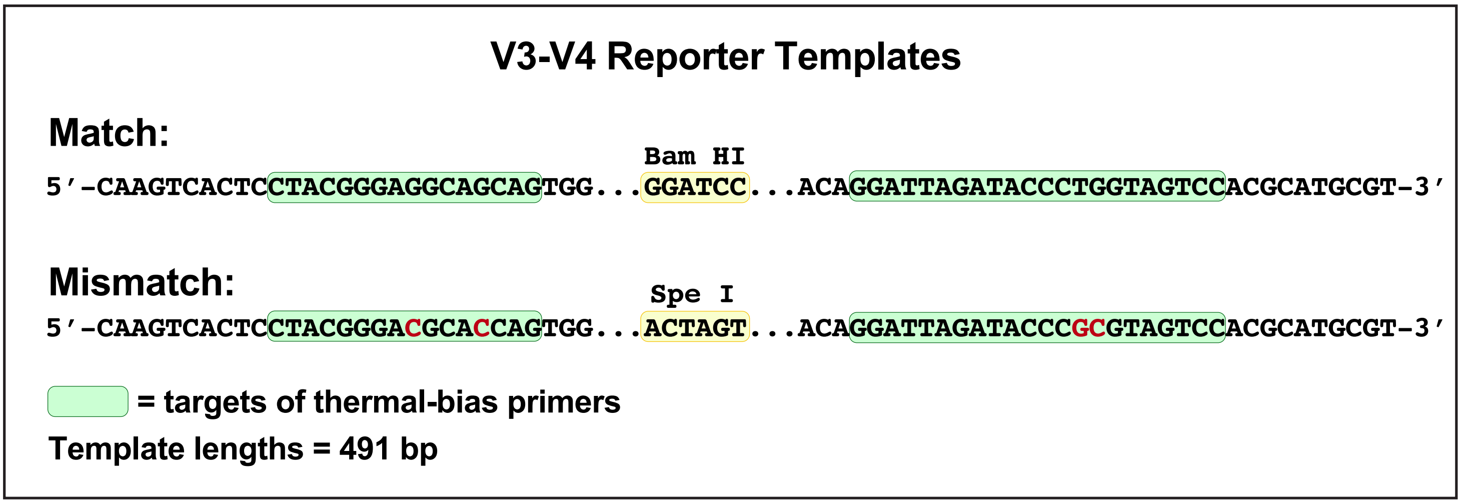

S3 Figure. Engineered V3-V4 reporter templates. Shown are the nucleotide sequences of the top strands of the 'match' and 'mismatch' reporters. Each has a unique restriction site inserted. The mismatch version has four nucleotide changes (red) in the primer-binding regions that are in positions predicted to be be compensated by the degenerate primers.
