# Supplementary material for: Thermal-bias PCR: generation of amplicon libraries without degenerate primer interference
Source: PeerJ. 2025 Oct 24;13:e20241. doi: 10.7717/peerj.20241 (PMC12558157; doi:10.7717/peerj.20241)
Supplement: Supplemental Information 5 — Non-degenerate primer pairs were evaluated for their ability to co-amplify the mismatch V3-V4 template (MM) in the presence of an equal amount of the match template (M). The non-degenerate pair ND_F1 and ND_F2_Tm54 and ND_R1 had matching Tms of ~54 °C (left set, lanes 2-4); the non-degenerate pair ND_F1 and ND_R2_Tm57 had matching Tms of ~57 °C (right set, lanes 5-7). PCRs used Platinum II Taq polymerase and the EMP cycling protocol. Purified PCR products were digested with SpeI to detect the mismatch amplicon. The mismatch amplicon was detected in the mixed template reactions (lanes 4 and 6). [file peerj-13-20241-s005.pdf]

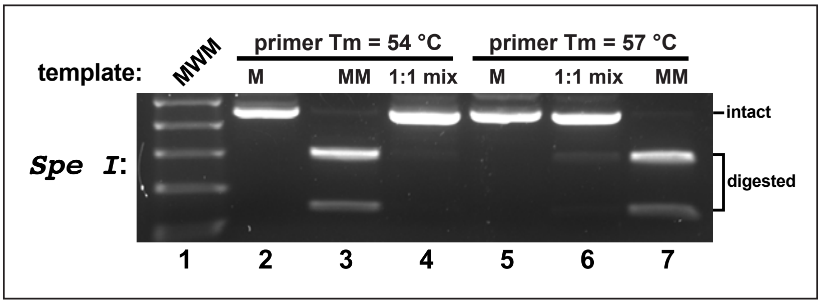

S4 Figure. Matching Tms improve production of a mismatch amplicon. Non-degenerate primer pairs were evaluated for their ability to co-amplify the mismatch V3-V4 template (MM) in the presence of an equal amount of the match template (M). The non-degenerate pair ND_F1 and ND_F2_Tm54 and ND_R1 had matching Tms of ~54 °C (left set, lanes 2-4); the non-degenerate pair ND_F1 and ND_R2_Tm57 had matching Tms of ~57 °C (right set, lanes 5-7). PCRs used Platinum II Taq polymerase and the EMP cycling protocol. Purified PCR products were digested with SpeI to detect the mismatch amplicon. The mismatch amplicon was detected in the mixed template reactions (lanes 4 and 6).
