# Supplementary material for: Thermal-bias PCR: generation of amplicon libraries without degenerate primer interference
Source: PeerJ. 2025 Oct 24;13:e20241. doi: 10.7717/peerj.20241 (PMC12558157; doi:10.7717/peerj.20241)
Supplement: Supplemental Information 6 — (A) SsoFast amplification of V3-V4 from E. coli genomic DNA (1.56 μg/μL) using nondegenerate or degenerate primers (ND_F1/R1 or Pro_341F/Pro_805R); 50 °C annealing/extension 60 sec. (B) Thermal-bias PCR using either SsoFast or Platinum Taq with either a match or mismatch engineered template (primers TB_F_Tm55_v1/TB_R_Tm55_v1). The Platinum Taq reactions were supplmented with EvaGreen. Targeting cycles used 50 °C annealing/extension for 5 min, amplification cycles used 80 °C annealing/extension for 1 min. [file peerj-13-20241-s006.pdf]

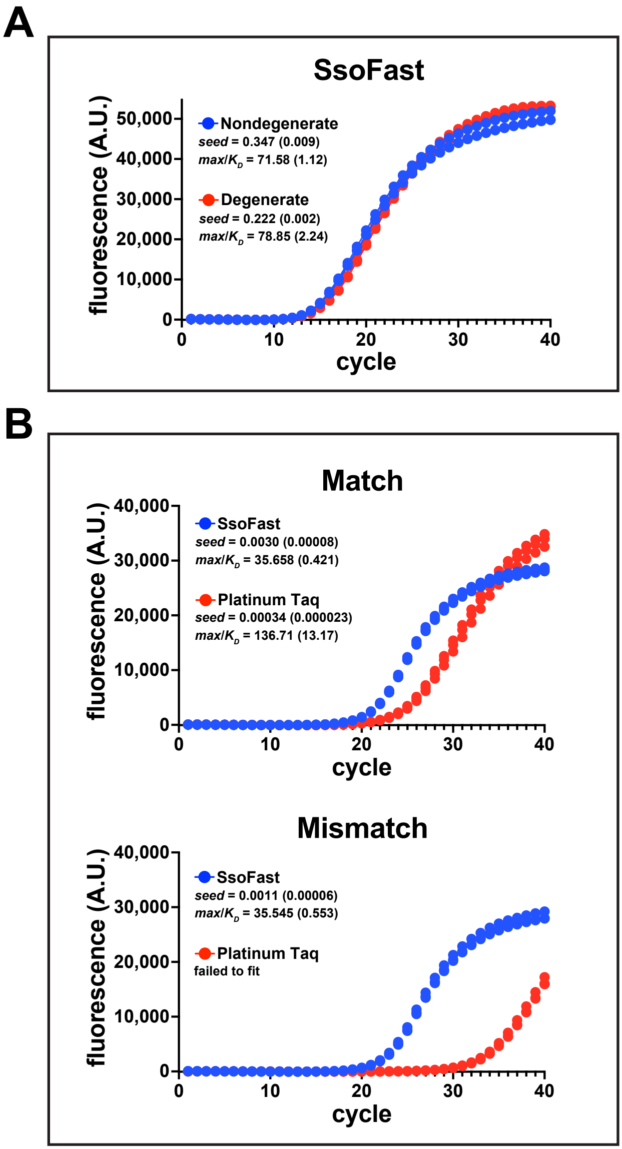

S5 Figure. Degenerate tolerance and thermal-bias PCR performance. (A) SsoFast amplification of V3-V4 from *E. coli* genomic DNA (1.56 µg/µL) using nondegenerate or degenerate primers (ND_F1/R1 or Pro_341F/Pro_805R); 50 °C annealing/extension 60 sec. (B) Thermal-bias PCR using either SsoFast or Platinum Taq with either a match or mismatch engineered template (primers TB_F_Tm55_v1/TB_R_Tm55_v1). The Platinum Taq reactions were supplmented with EvaGreen. Targeting cycles used 50 °C annealing/extension for 5 min, amplification cycles used 80 °C annealing/extension for 1 min.
