# Supplementary material for: Thermal-bias PCR: generation of amplicon libraries without degenerate primer interference
Source: PeerJ. 2025 Oct 24;13:e20241. doi: 10.7717/peerj.20241 (PMC12558157; doi:10.7717/peerj.20241)
Supplement: Supplemental Information 7 — A) The match and mismatch V3-V4 templates were used to evaluate PCR performance at various elevated amplification temperatures. Two targeting cycles were performed at 50 °C and 40 amplification cycles were performed at the indicated annealing temperatures. B) The match template was used in a PCR protocol lacking the two targeting cycles, but contained 30 amplification cycles at the indicated temperatures. The absence of targeting strongly suppressed amplification at 74 °C (~11 cycles, ~2000-fold), and abolished amplification at 78 °C and 80 °C. These experiments used SsoFast polymerase and the primer targeting sections had calculated Tms of 55 °C and they contained Illumina TruSeq adapter sequences in their tails (TB_F_Tm55_v2 and TB_R_Tm55_v2). [file peerj-13-20241-s007.pdf]

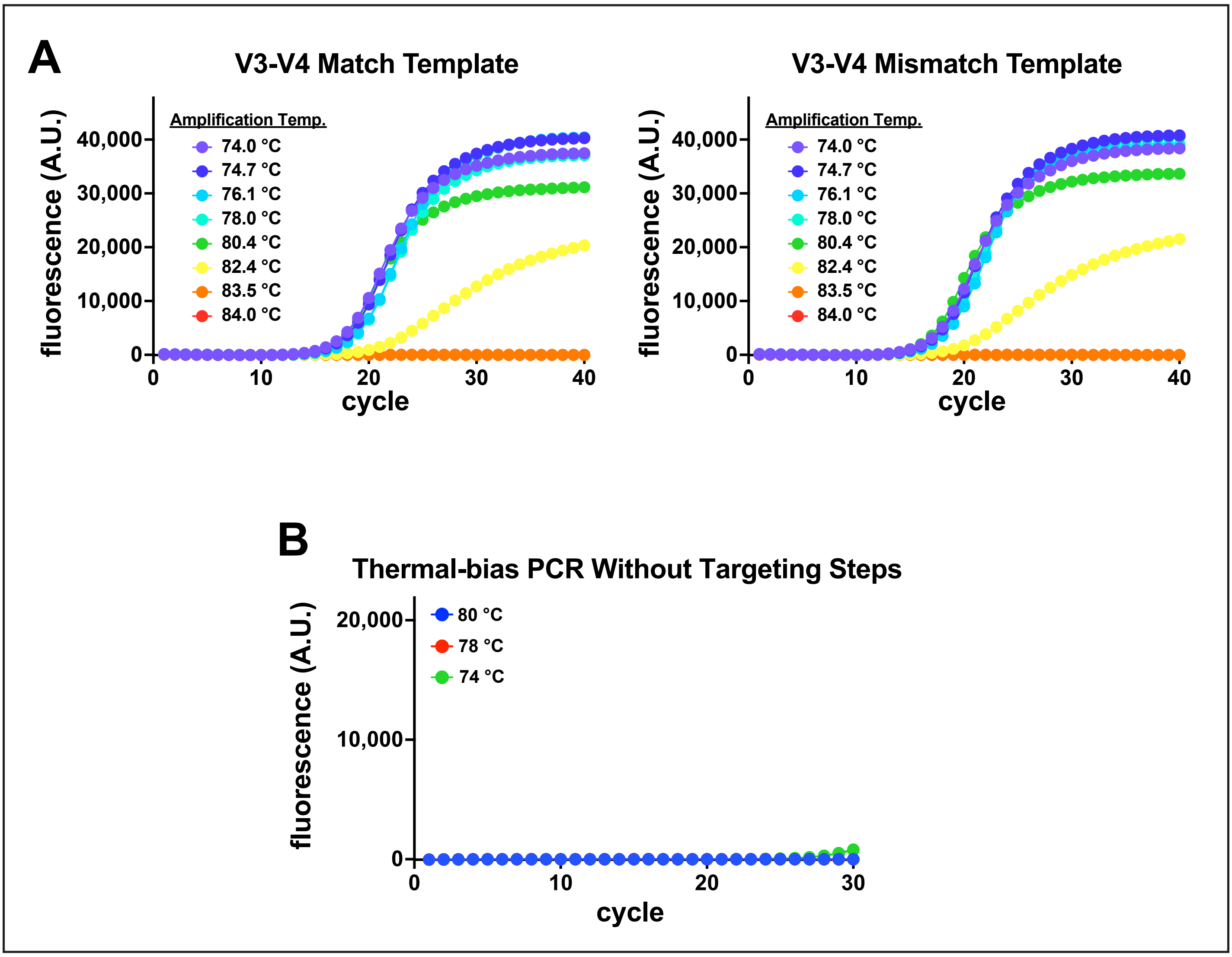

S6 Figure. Amplification and target-priming thermal-bias. A) The match and mismatch V3-V4 templates were used to evaluate PCR performance at various elevated amplification temperatures. Two targeting cycles were performed at 50 °C and 40 amplification cycles were performed at the indicated annealing temperatures. **B)** The match template was used in a PCR protocol lacking the two targeting cycles, but contained 30 amplification cycles at the indicated temperatures. The absence of targeting strongly suppressed amplification at 74 °C (~11 cycles, ~2000-fold), and abolished amplification at 78 °C and 80 °C. These experiments used SsoFast polymerase and the primer targeting sections had calculated Tms of 55 °C and they contained Illumina TruSeq adapter sequences in their tails (TB_F_Tm55_v2 and TB_R_Tm55_v2).
