# Supplementary figures and images for: Thermal-bias PCR: generation of amplicon libraries without degenerate primer interference
Source: PeerJ. 2025 Oct 24;13:e20241. doi: 10.7717/peerj.20241 (PMC12558157; doi:10.7717/peerj.20241)

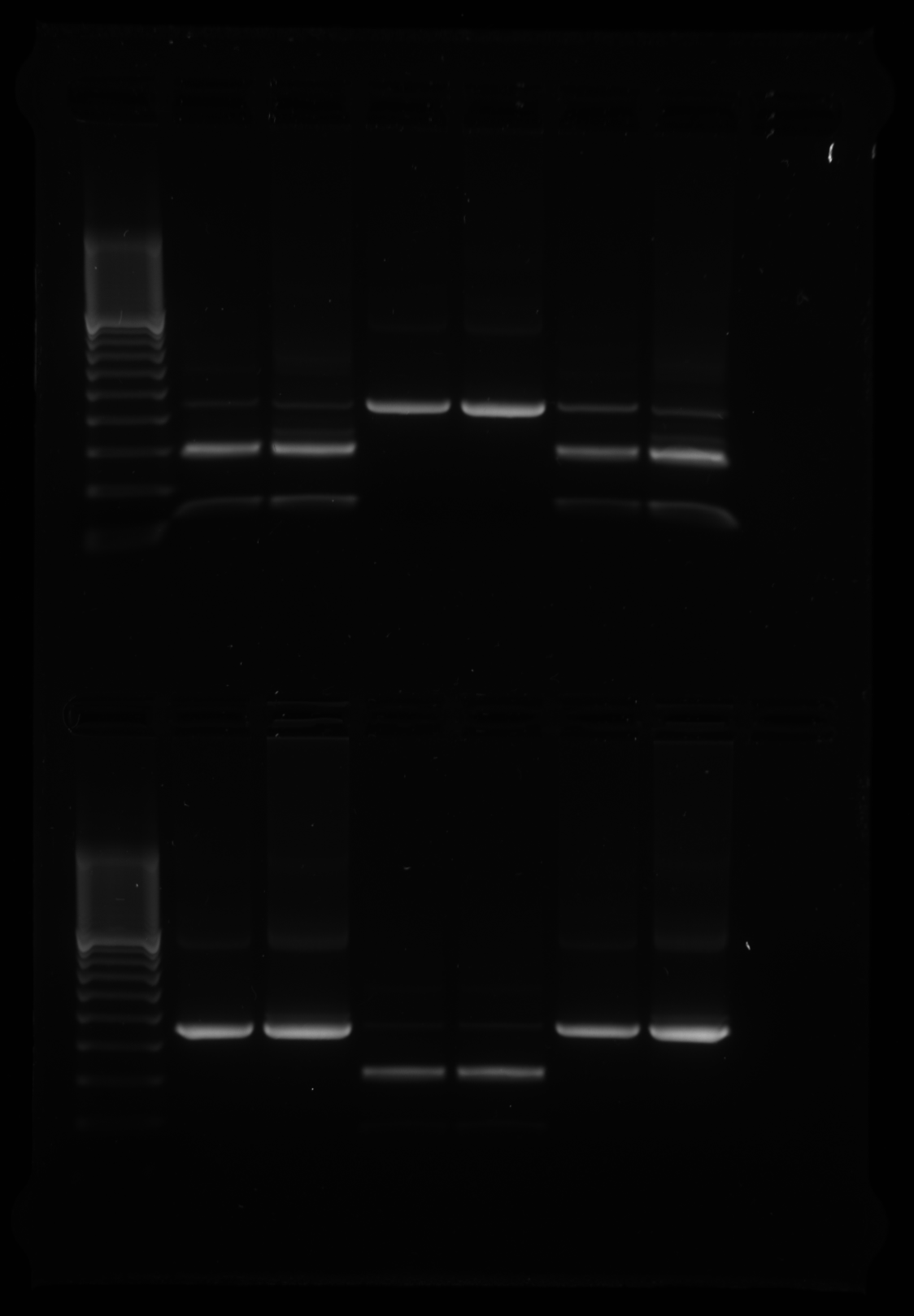

Supplement: Supplemental Information 8 — Full size gel images of figure panels 2C, 3C, and S4. [file peerj-13-20241-s008.zip › Fig 2C.raw16.png]

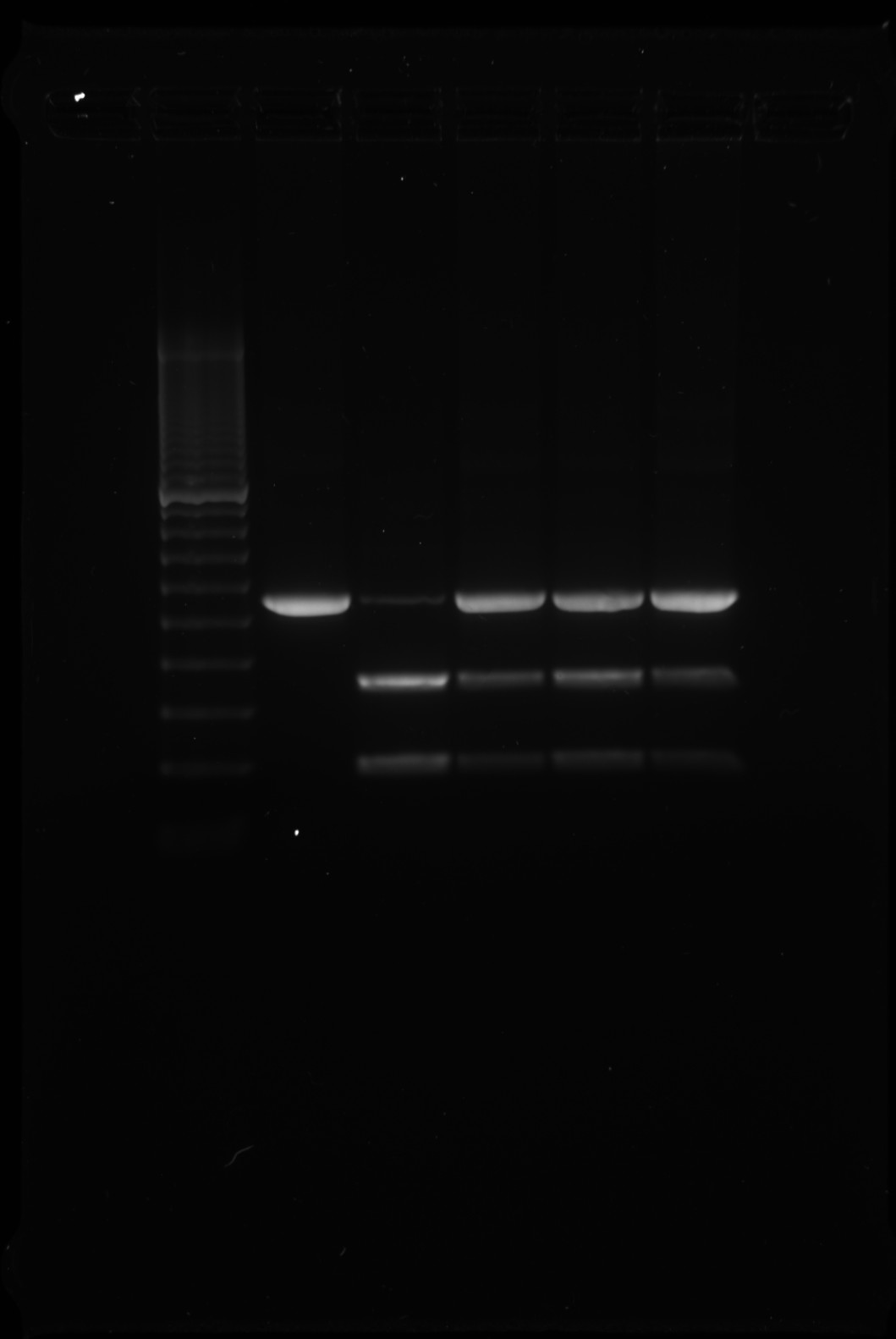

Supplement: Supplemental Information 8 — Full size gel images of figure panels 2C, 3C, and S4. [file peerj-13-20241-s008.zip › Fig 3C.raw16.png]

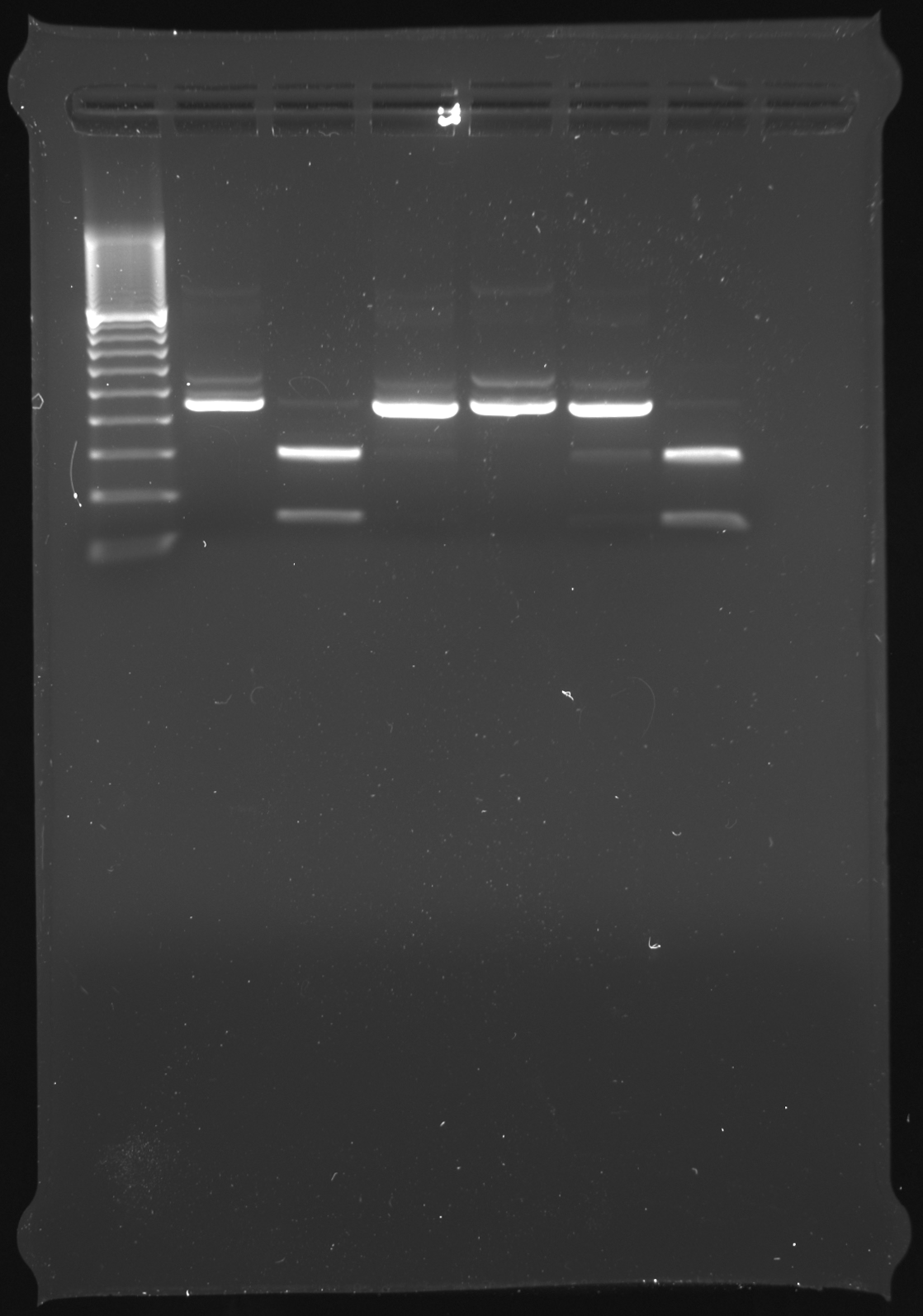

Supplement: Supplemental Information 8 — Full size gel images of figure panels 2C, 3C, and S4. [file peerj-13-20241-s008.zip › Fig S4.raw16.png]
